# Lung cancer epidemiology in North Sardinia, Italy

**DOI:** 10.1186/2049-6958-8-45

**Published:** 2013-07-12

**Authors:** Panagiotis Paliogiannis, Federico Attene, Antonio Cossu, Mario Budroni, Rosaria Cesaraccio, Francesco Tanda, Mario Trignano, Giuseppe Palmieri

**Affiliations:** 1 Department of Surgical, Microsurgical and Medical Sciences, University of Sassari, V. le San Pietro 43B, 07100, Sassari, Italy; 2 Service of Epidemiology, A.S.L. 1, Via Amendola 55, 07100, Sassari, Italy; 3 Institute of Biomolecular Chemistry, Cancer Genetics Unit, C.N.R., TraversaLa Crucca 3, 07040, Sassari, Italy

**Keywords:** Adenocarcinoma, Italy, Lung cancer, Sardinia, Screening, Small cell, Squamous

## Abstract

**Background:**

The aim of this study was to analyze and describe the epidemiological characteristics and trends of lung cancer in North Sardinia, Italy, in the period 1992–2010.

**Methods:**

Data were obtained from the tumor registry of Sassari province which is a part of a wider registry web, coordinated today by the Italian Association for Tumor Registries.

**Results:**

The overall number of lung cancer cases registered was 4,325. The male-to-female ratio was 4.6:1 and the mean age 68.1 years for males and 67 years for females. The standardized incidence rates were 73.1/100,000 and 13.5/100,000 and the standardized mortality rates 55.7/100,000 and 9.9/100,000 for males and females, respectively. An increasing trend in incidence of lung cancer in women was evidenced. Conversely, incidence was found to decrease in males. Relative survival at 5 years from diagnosis was low (8.8% for males and 14.9% for females). Furthermore, an increase in mortality rates was observed in both sexes in the period under investigation.

**Conclusions:**

Our data show an increasing trend of lung cancer incidence in women in North Sardinia in the last decades. Conversely, a reduction of incidence rates was observed in males. Furthermore, a slightly increasing trend in mortality rates was observed in both sexes, suggesting the need to enhance smoking control strategies, consider adoption of effective surveillance policies, and improve diagnosis and treatment methods.

## Background

Lung cancer is the most common neoplastic disease in the world with more than 1,600,000 cases estimated in 2008 [1]. Considering world incidence rates**,** lung cancer ranks first in men and fourth in women after breast, cervix uteri and colorectal cancer [1]. It also represents the most frequent neoplastic cause of death worldwide [1]. A consistent increase in lung cancer incidence was recently registered in developing countries and in women, while it began to decline in males in most Western countries [2,3]. These trends reflect primarily the continuous changes in smoking habits and environmental pollution, particularly in developing countries, as well as the progressive improvement of life expectancy in Western countries. Since the first relevant publications in the fifties of the past century, it has been evidenced that smoking is the risk factor that mostly impacts the epidemiological trends of lung cancer [4,5]. Even if other factors such as workplace exposure to pollution and genetic determinants were evidenced to contribute to lung carcinogenesis, active and passive smoking remain the most incident risk factor. This induced the adoption of smoking control campaigns, especially in developed countries, which recently lead to progressive reduction of lung cancer incidence and mortality in males in these countries.

The aim of this population-based study was to analyze and describe the epidemiological characteristics and trends of lung cancer in north Sardinia, Italy, in the period 1992–2010.

## Methods

The epidemiological data presented in this article were obtained from the “Registry of the tumors of the Province of Sassari”. This registry was created in 1992 by the local health agency for the epidemiological surveillance of tumors in that province. In 1999 it became part of a wider web of tumor registries, coordinated today by the Italian Association for Tumor Registries (AssociazioneItalianaRegistriTumori, AIRTUM). The association coordinates 34 registries in the country, collects and publishes data, and collaborates with international organizations in the field.

Every registry collects data on tumoral diseases affecting inhabitants in the territory of jurisdiction through the local hospitals and health care services, as with other registries (e.g., death registries). Demographic, clinical, pathological and prognostic data are collected for each case of cancer and are registered in a digital database. This database was the data source for the present population-based report.

The demographic characteristics of the patients affected by lung cancer were collected. Crude incidence and mortality rates per 100,000 inhabitants per year were calculated, and the standardized rates were adjusted for European age-population standards. Furthermore, time trends of incidence rates by age class and gender were studied, as well as the estimated change in annual percentage. A comparison between incidence and mortality in the province of Sassari and those in other Italian provinces was also performed. In addition, the cumulative risk of developing the disease and of dying between zero and 74 years of age was estimated. The age class distribution and time trends of incidence, mortality, mean age of disease onset and death, and histology were evaluated. Finally, relative 5-year survival was calculated by the Hakulinen method.

## Results

The overall number of cases of lung cancer registered in the period 1992 – 2010 was 4,325. The diagnosis was obtained by histological or cytological reports in 3,178 cases (73.5%) and using other information sources (clinical reports, radiological referrals, death certifications, etc.) in 1,129 cases (26.1%). The modality of diagnosis was not known in 18 cases (0.4%). Among the 4,284 individuals registered, 3,554 were males and 771 females, with a male-to-female ratio of 4.6:1. The mean age was 68.1 years for males and 67 years for females. The cumulative risk of developing the disease was 6.13% for males and 1.11% for females.

**Figure 1:**
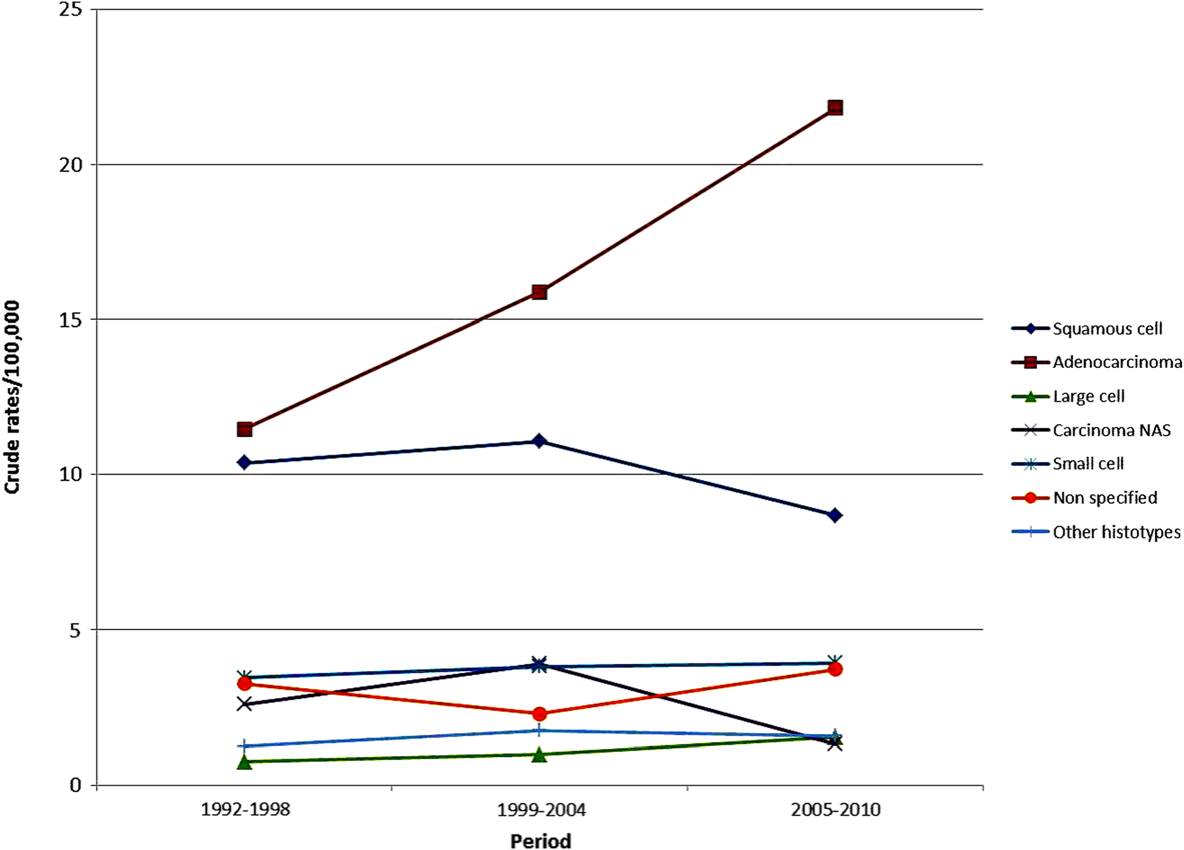
Trends in histological subtypes of lung cancer in North Sardinia, 1992–2010.

With regard to the anatomical distribution of the tumors 568 (13.1%) were located in the tracheo-bronchial tree, 1,493 (34.5%) in the upper lobes, 231 (5.3%) in the right middle lobe, 781(18.1%) in the inferior lobes and 272 (6.3%) in more than one lobe, while in 980 (22.7%) cases the anatomical localization was not known. Among the 3,178 tumors that had histological or cytological diagnosis, 1,330 (41.9%) were adenocarcinomas, 845 (26.6%) were squamous cell carcinomas, 310 (9.8%) were small cell cancers, 88 (2.8%) were large cell cancers and 126 (4%) were other histotypes, while in the remaining 479 (15%) cases the exact histologic type was not specified. The percentages of adenocarcinomas were 42.2% and 40.1% respectively in males and females with known histology, while the corresponding figures for squamous cell carcinomas were 26.4% and 27.4%. Furthermore, no significant differences in the distribution of other rarer histological subtypes among sexes were found. Figure 1 depicts the global trends of the principal histotypes in the years under investigation.

The crude incidence of lung malignancies in the period under investigation was 86.5/100,000 for men and 18.1/100,000 for women. Standardized incidence rates were 73.1/100,000 for males and 13.5/100,000 for females.

**Figure 2:**
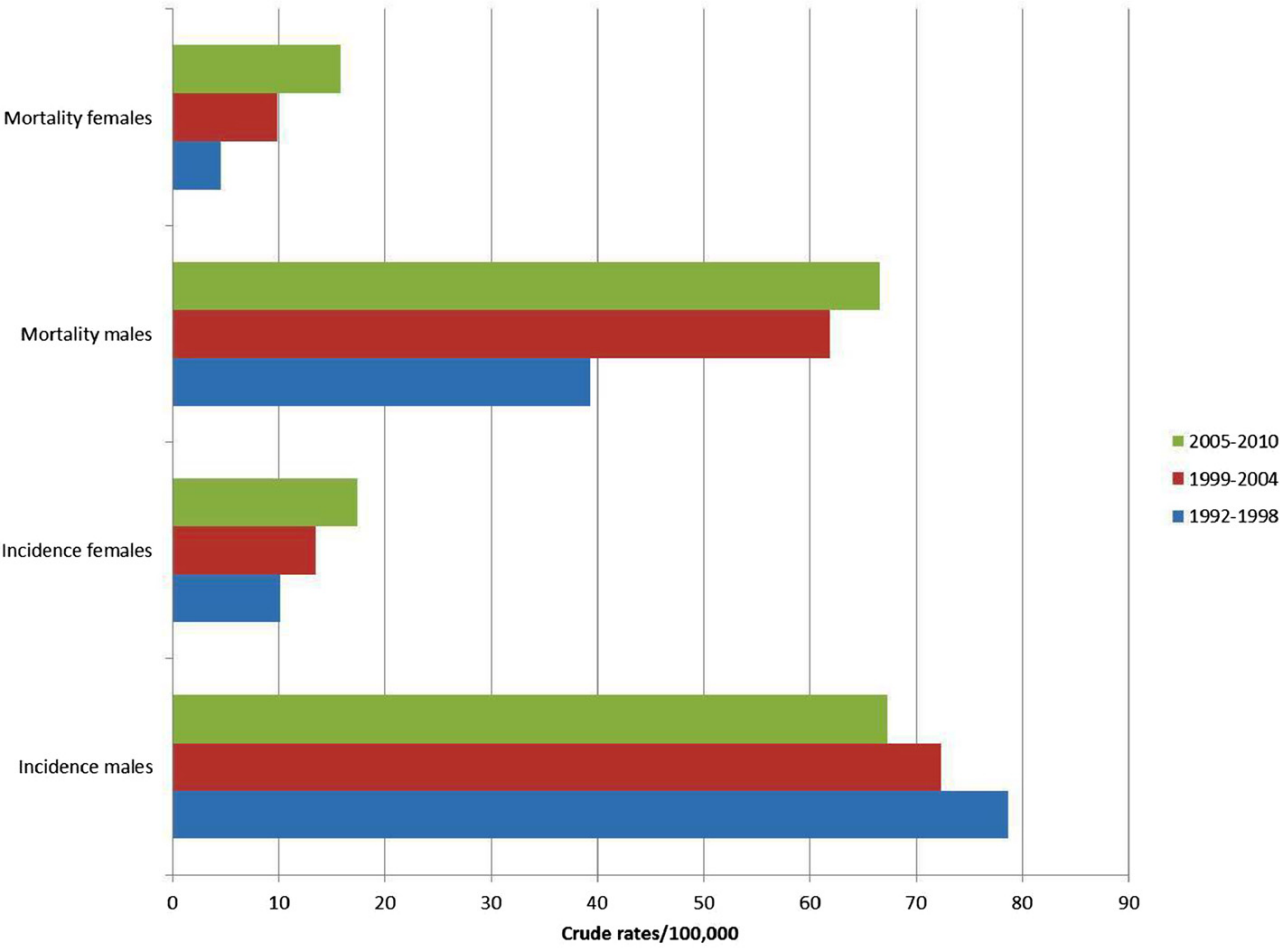
Incidence and mortality rates trends of lung cancer in North Sardinia, 1992–2010.

**Figure 3:**
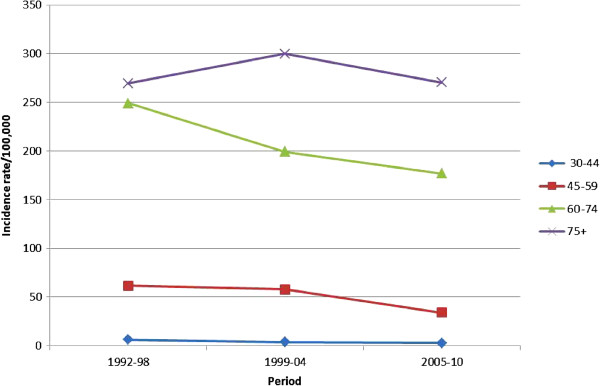
Age-class incidence rates trends of lung cancer in males in North Sardinia, 1992–2010.

**Figure 4:**
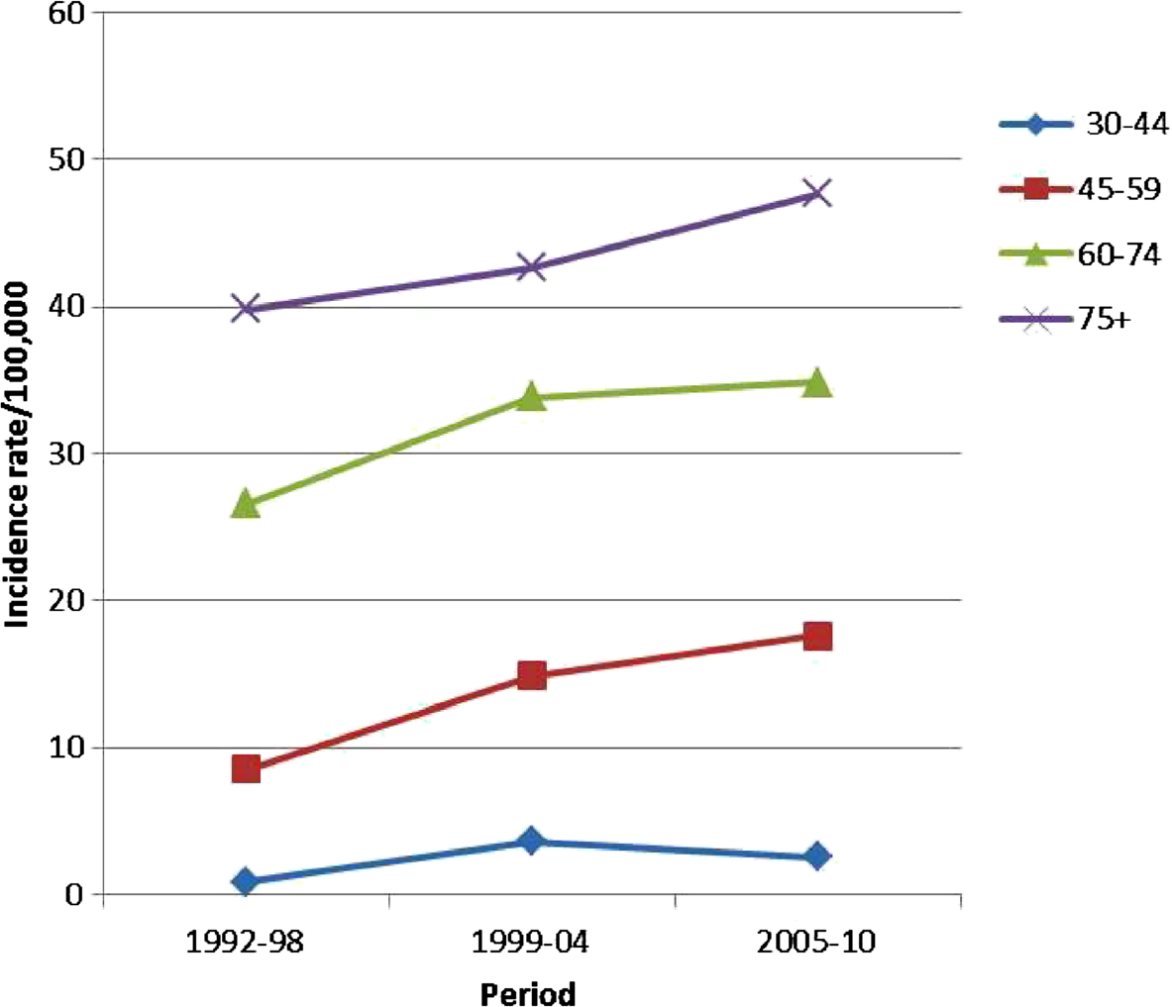
Age-class incidence rates trends of lung cancer in females in North Sardinia, 1992–2010.

Table 1 shows the percent distribution of incidence in relation to age, while Table 2 shows the distribution of incidence rates in relation to age. Peak incidence occurred at 75–79 years for both males and females. Incidence rates were also calculated for the following three time periods: 1992–1998, 1999–2004 and 2005–2010 (Figure 2). There was a progressive decrease in incidence rates in males, from 78.7/100,000 in the first period, to 72.3/100,000 in the second period and 67.3/100,000 in the last period. The corresponding figures for females were 10.1/100,000, 13.5/100,000 and 17.4/100,000, respectively. Figures 3 and 4 illustrate the trends of incidence by age-class and gender. A constant increase in incidence occurred between 1992 and 2010 in women, especially those older than 45 years. No significant changes in incidence were evidenced in younger age groups in both sexes. The estimated annual percentage change in males was −0.19, −1.53, -4, and −0.04 for 30–44, 45–59, 60–74, ≥75 age groups respectively. The corresponding figures in women were 0.09, 0.5, 0.46, and 0.44. Analysis of the trend of mean age at disease onset for the same periods of time did not reveal any relevant changes. Table 3 shows the comparison of the incidence and mortality in the province of Sassari with those in other Italian provinces.

**Table 1:** Age-class incidence distribution of lung cancer in North Sardinia, 1992-2010

**Age (years)**	**Number of cases (%)**	
	**Males**	**Females**
0-14	0 (0)	0 (0)
15-29	2 (0.06)	2 (0.26)
30-44	60 (1.69)	33 (4.28)
45-59	656 (18.46)	173 (22.44)
60-74	1,819 (51.18)	315 (40.86)
75+	1,017 (28.62)	248 (32.17)

**Table 2:** Age-class incidence and mortality rates of lung cancer in North Sardinia, 1992–2010

**Age (years)**	**Incidence (/100,000 per year)**		**Mortality (/100,000 per year)**	
	**Males**	**Females**	**Males**	**Females**
0-4	0	0	0	0
5-9	0	0	0	0
10-14	0	0	0	0
15-19	0	0	0	0
20-24	0	0.4	0	0
25-29	0.6	0.3	0	0
30-34	1.8	1.8	1.5	0.6
35-39	3.9	3.9	2.1	1.5
40-44	12.9	4.4	6.9	3.5
45-49	37.7	15.3	20.4	9.2
50-54	72.5	19.4	50.6	14.6
55-59	141.2	29.2	100,4	16.7
60-64	240.4	35.5	168.4	25.5
65-69	329.6	50.5	229.8	36.4
70-74	424.4	62.5	334.5	47.8
75-79	495.3	85.8	442.9	73.1
80-84	472	74	453.2	74.9
85+	298.2	44.4	290.2	50.9
**Total**	**86.5**	**18.1**	**67**	**14**

**Table 3:** Comparison with incidence and mortality rates of other Italian provinces, 1992-2010

**Province**	**Incidence (/100,000 per year)**		**Mortality (/100,000 per year)**	
	**Males**	**Females**	**Males**	**Females**
**Alto Adige**	59	16.2	51.9	12.7
**Biella**	94	16.3	80.1	14
**Ferrara**	96.8	18.7	81.7	16.2
**Florence**	77.4	17.1	65.5	12.7
**Friuli V.G.**	78.8	19.2	71.5	16
**Genoa**	97.9	16.6	80.1	12.6
**Macerata**	64	11.9	57	8
**Modena**	82.9	18.6	72.9	15.4
**Naples**	94.8	13	83	10.6
**Parma**	80.8	20	65.2	14.9
**Ragusa**	65	8.4	56.6	7.3
**Reggio Emilia**	76.9	19	69.9	14.5
**Romagna**	87.7	20.3	71.5	14.5
**Salerno**	74.3	8.8	66.1	7.6
**Sassari**	**73.1**	**13.5**	**55.7**	**9.9**
**Turin**	87.9	18.2	75.7	14.2
**Trento**	68	11.8	66.6	10.7
**Umbria**	66.7	14.2	55.4	9.8
**Varese**	84.1	14.5	81.1	12
**Veneto**	95.1	21.1	85.7	15.5
**Mean**	**80.8**	**16.9**	**69.9**	13.1

There were 3,347 deaths in the period under investigation (2,751 males and 596 females). Crude overall mortality was 67/100,000 for males and 14/100,000 for females. Mean age at death was 69.4 years in males and 69.2 years in females. Standardized mortality rates were 55.7/100,000 for males and 9.9/100,000 for females. The cumulative risk of death was 4.47% for males and 0.78% for females. Table 2 shows the age-class distribution of mortality rates. There was a relevant increase in mortality rates after the fifth decade of life. Figure 2 shows the time trend of mortality between 1992 and 2010: a significant increase in mortality in both sexes was registered. Finally, relative survival at 5 years from diagnosis was 10% (8.8% for males and 14.9% for females). Relative 5-years survival was 9.3% in the period 1992–1998, 10.4% in the period 1999–2004, and 10.3% in the last period under investigation (2005–2010).

## Discussion

World population age-standardized incidence of lung cancer calculated in 2008 was 22.9/100,000 (33.8/100,000 for men and 13.5/100,000 for women), while cumulative risk was 2.77% [1]. Lung cancer is also the most frequent cause of death for cancer, with more than 1,370,000 deaths in the world in the same year [1].

The incidence of lung cancer is significantly higher in most industrialized areas worldwide in comparison with developing countries; however, these countries present continuously increasing incidences in the last decades [1,6]. In Europe more than 380,000 cases of lung cancer were estimated in 2008, with a standardized incidence rate of 48.9/100,000 for men and 13.4/100,000 for women, and approximately 340,000 deaths, with standardized mortality rates of 42.5/100,000 for males and 10.8/100,000 for females. The highest figures were observed in central and eastern European countries (Hungary and Poland) [1].

In Italy 38,500 new cases and 34,500 deaths for lung cancer were estimated in 2012 [7]. Different incidence figures were observed throughout the country in the period 2006–2008: 72.9/100,000 in northern, 63.8/100,000 in central and 65.4/100,000 in southern regions [7]. The standardized incidence rates in the province of Sassari were similar to those estimated by AIRTUM for northern Italian regions. Comparisons of the incidence rates with those of other Italian provinces or regions place our province in the middle between those with low incidence rates, such as Alto Adige and Macerata, and those with higher incidences like Genoa, Ferrara and Veneto (Table 3). Lung cancer incidence rate was lower in North Sardinia than average rate in Italy; this may be due to a minor exposure to occupational and environmental carcinogens like asbestos, arsenic, radon and polycyclic aromatic hydrocarbons, given the lower diffusion of industrial activities in the area in the last decades, in comparison to other Italian provinces [8].

Concerning histology, our data evidenced a prevalence of adenocarcinomas over squamous cell carcinomas and other subtypes of lung cancer. Furthermore, a decline in incidence of squamous cell carcinomas was evidenced, as opposed to other histological types which presented a slight increase in incidence rates between 1992 and 2010. A remarkable shift in world lung cancer incidence rates by histologic subtype occurred in the last decades. Squamous cell carcinoma was the most frequently observed histotype in the initial epidemiological reports since the 80’s when it was superseded by adenocarcinoma [2,9]. The causes of such shifting are not clear and several hypotheses have been proposed, concerning mainly factors linked to tobacco smoking: changes in the characteristics of cigarettes, increased puff volume, increased nitrate levels and, finally, higher incidences in women who tend to have adenocarcinomas more frequently than parallel male cohorts [10,11]. This tendency was not confirmed in our cohort in which adenocarcinomas and squamous cell carcinomas involved both sexes similarly, but a relatively high percentage of cases without histological diagnosis was also observed (15%), due to some procedural limitations in data collection and registration.

Considering the distribution of the disease in relation to age, less than 5% of the cases occurred in individuals <45 years, while more than 75% occurred after the sixth decade of life. This reflects the modalities of exposure to risk factors like smoking and pollution and the long latency from exposure to disease. Incidence rates increased with aging in both sexes, reaching peak values in individuals ≥ 80 years. Incidence rates trends remained stable in individuals <45 years, decreased in all age groups in males ≥45 years (especially in the last decade), and increased in the corresponding age groups in females. The study of the estimated annual percentage change by age group demonstrated that incidence rate reductions in males and increases in females involved mainly individuals between the fifth and seventh decade of life. This distribution pattern is similar to those reported for the world population [6].

The time trends analysis showed a steady increase in incidence of lung cancer in females in Sassari province in the period under investigation. Conversely, a slight reduction in incidence rates was observed in males. These trends are common to numerous national and international geographical areas, and may reflect the increasing diffusion of tobacco smoking in women as opposed to the reduction of smoking incidence in men. The world standardized incidence rates of lung cancer increased by 22% among females and decreased by 3% among males in the period 1985–2002 [12]. Giving the current smoking trends, it is calculated that by 2030 lung cancer will affect both sexes equally [2].

The role of screening programs in patients with risk factors for lung cancer is still a matter of debate. Some studies, especially those performed using standard chest X-ray, did not reveal any impact of the screening program on survival. More recent contributions, with a longer follow up time available and better technologies employed, showed a relevant decrease in mortality rates in enrolled patients, and posed the question of the utility of screening strategies in lung cancer prevention [13]. Nevertheless, several aspects remain to be addressed before the introduction and diffusion of screening campaigns in clinical practice. To date no screening programs for lung cancer are active in North Sardinia, as opposed to numerous smoking control campaigns.

Concerning mortality, 3,347 (2,751 males and 596 females) deaths occurred in the 18 years we studied. Standardized mortality rates were considerably inferior in women. Considering the age-class mortality trend, a natural increase in relation to age was observed in both sexes, with peaks after the eighth decade of life and with a slight increase between 1992 and 2010 (Table 2). Standardized mortality rates increased in Sassari province in the years under investigation, as opposed to global national figures which evidenced a steady decrement of mortality rates in males in the last 2 decades (−2.2% per year) and a continuous increment in females (+1% per year) [7].

Finally, the relative 5 years survival from diagnosis was low in both sexes (10%), but in accordance with percentages published for other developed countries and for the entire country [14]. Several factors impact on such low survival in patients with lung cancer such as the lack of effective screening programs, non-specific clinical manifestations, delays in diagnosis, high percentage of advanced stages at diagnosis, smoking related comorbidities, ineffectiveness of current therapeutic strategies, and others. The relative 5 years survival was better in women, but the gap observed between sexes will probably decrease in the future as a consequence of the steadily increasing smoking incidences in women [2,9]. The current gap may depend on several factors, other than smoking habits, like biological dissimilarities or differences in histology, stage and therapy. Globally, the relative 5-years survival improved in our cohort from 1992 to 2010.

## Conclusions

Our data showed an increasing trend in incidence of lung cancer in women in North Sardinia in the last decades. Conversely, a reduction of incidence rates was observed in males. Furthermore, a slightly increasing trend in mortality rates was observed in both sexes, suggesting the need to enhance smoking control strategies, consider adoption of effective surveillance policies**,** and improve diagnosis and treatment methods.

## Competing interests

The authors declare that they have no competing interests.

## Authors’ contributions

PP, FA, AC and MB contributed to design the study, write the manuscript and revise editing. MB and RC contributed to the collection and analysis of statistical data. MB, RC, FT and MT contributed to the interpretation of data and revision of the manuscript. GP performed the final revision of the manuscript. All authors read and approved the manuscript.
